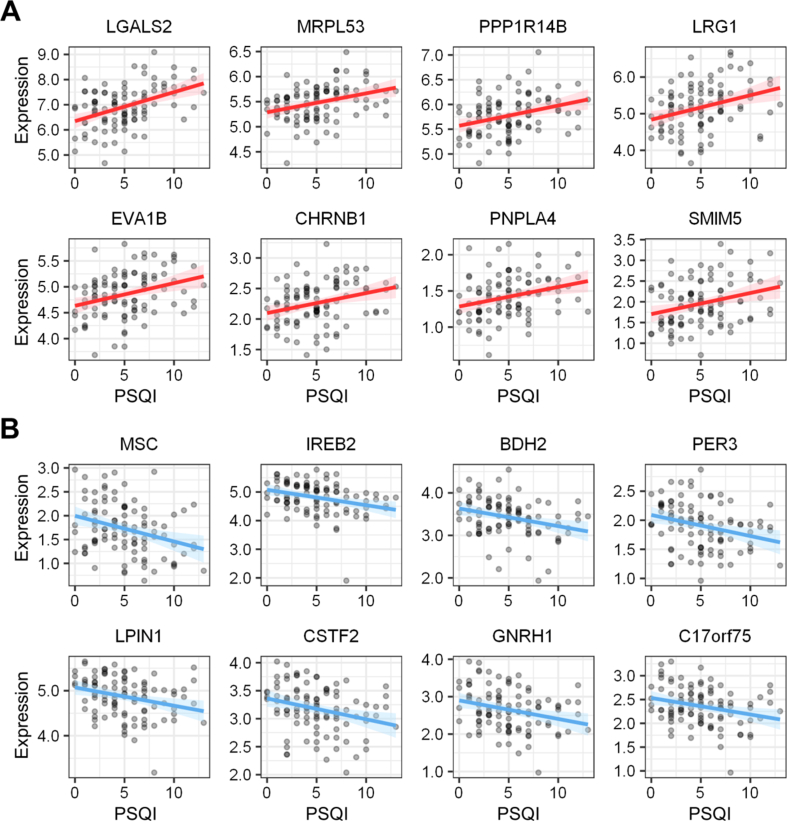# Blood transcriptomic markers associated with immune abnormalities and sleep quality

**DOI:** 10.1016/j.gendis.2023.101105

**Published:** 2023-09-14

**Authors:** Sang-Min Park, Hyo-Jeong Ban, Minsung Lee, Soo Yeon Kim, Siwoo Lee, Hee-Jeong Jin

**Affiliations:** a College of Pharmacy, Chungnam National University, Daejeon 34134, Republic of Korea; b Korean Medicine (KM) Data Division, Korea Institute of Oriental Medicine, Daejeon 34054, Republic of Korea

The prevalence of sleep disorders is increasing worldwide, prompting greater efforts to understand the biological mechanisms underlying sleep. Despite the growing interest in this area, a continuous transcriptome analysis for studying sleep quality is still lacking. This study performed a regression analysis of the Pittsburgh sleep quality index (PSQI) from the blood transcriptome to identify marker genes associated with sleep quality. In this cohort study, sleep quality scores were defined for 100 participants, and molecular properties associated with the scores were analyzed using blood transcriptome analysis. Gene expression and pathway enrichment analyses revealed that immune-related dysregulation was prominently associated with sleep quality. To understand the genetic influences on our observations, a genome-wide association study (GWAS) was conducted to examine the relationship between sleep quality and immune indicators. The results revealed a relationship between sleep and the immune system and confirmed a genetic correlation. Our study provides valuable insights into the complex interplay between the genetic and environmental factors that determine sleep quality and their impact on overall health.

This study included the same participants (*n* = 100) as our previous study^1^ for exploring genes associated with metabolic syndrome progression, selected from the Korean Medicine Daejeon Citizen Cohort (KDCC) study.^2^ To identify genes associated with sleep quality (Fig. S1), we performed a linear regression analysis between gene expression levels and PSQI. We identified 170 and 422 genes whose expression increased and decreased, respectively, as PSQI increased (*P* < 0.05, positive and negative β-values for PSQI) (Fig. 1A, left). Considering that RNA-seq data mostly follow a negative binomial distribution,^3^ we built a negative binomial regression model. We identified 158 and 280 genes whose expression increased and decreased, respectively, with increasing PSQI (*P* < 0.05, positive and negative β-values for PSQI) (Fig. 1A, right). The associations between gene expression and PSQI scores were consistent between linear and negative binomial regression models (Fig. 1B). From the common genes in the two models, we selected 153 and 274 genes with positive and negative associations with PSQI as markers of poor and good sleep quality, respectively (Table S1). Poor markers were defined as genes whose expression increased as sleep quality decreased, and *vice versa* for good markers.

**Figure 1:**
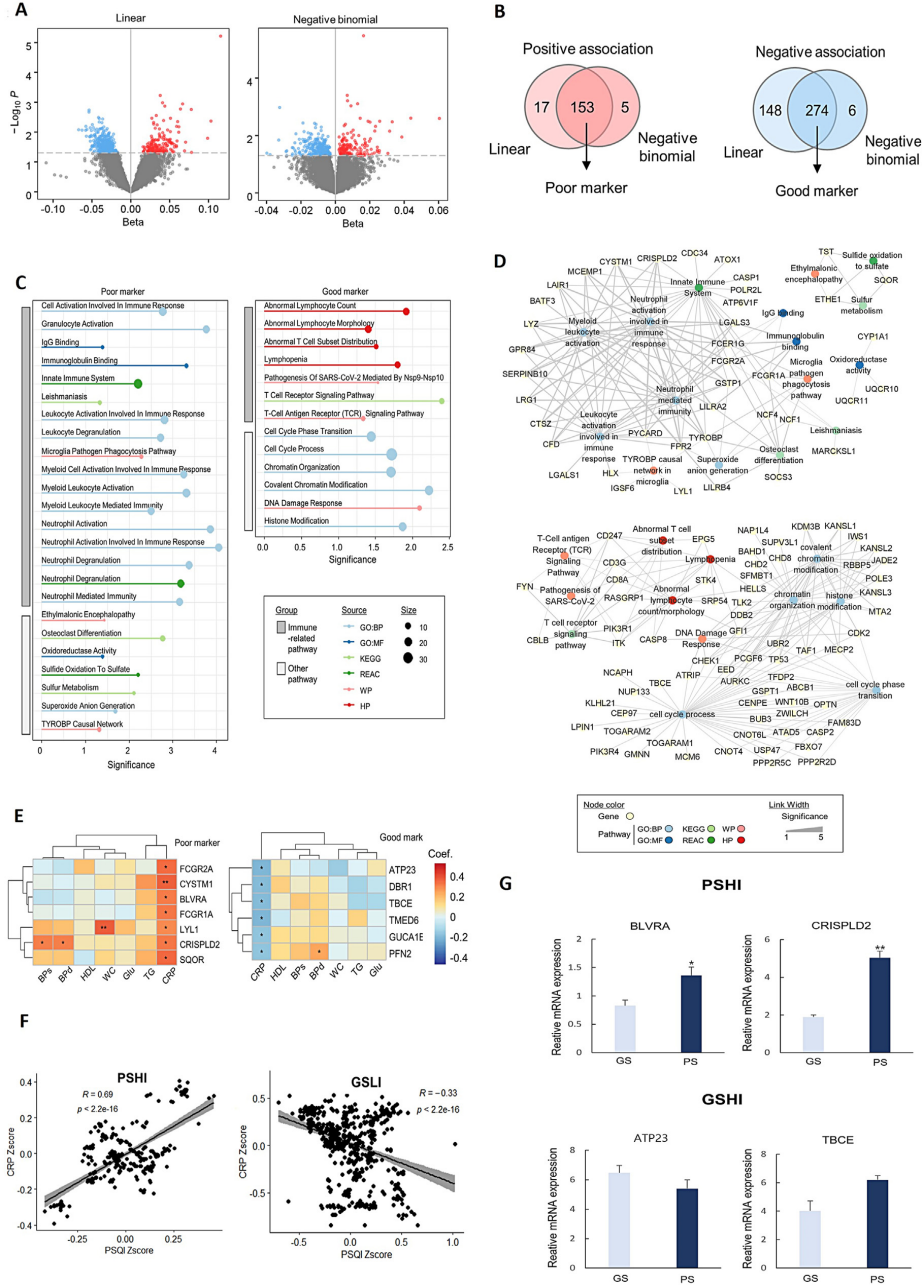
Blood transcriptomic markers associated with immune abnormalities and sleep quality. **(A)** Identification of the transcriptomic markers of sleep quality. Results of linear (left) and negative binomial (right) regression analysis for gene expression levels with Pittsburgh sleep quality index (PSQI) scores. **(B)** Number of significantly associated marker genes with PSQI scores. Expression levels of poor and good marker genes are positively and negatively associated with PSQI scores, respectively. **(C)** Pathway enrichment analysis for transcriptomic markers of sleep quality. Significantly enriched pathway terms for the poor (left) and good (right) markers of sleep quality. Significance is calculated as −log_10_(*P*). Source represents gene sets to which pathway terms belong, expressed as colors. Size represents intersection size, which is the number of common genes in marker groups and pathway terms, expressed as the size of the dots. **(D)** Network analysis for transcriptomic markers of sleep quality. Integrated functional networks for the poor and good markers with enriched pathways. Each marker gene is linked to associated enriched pathways. Node color denotes the source of the gene set involved in a pathway. The width of the link denotes the significance of enrichment analysis calculated as −log_10_(*P*). **(E)** Transcriptomic markers associated with both sleep quality and inflammation level. Genes significantly associated with C-reactive protein (CRP) in a positive or negative relationship with the poor (left) or good (right) markers, respectively. Correlation coefficients are indicated by color. ∗*P* < 0.05, ∗∗*P* < 0.01. **(F)** Genetic correlation between PSQI and CRP within poor sleep quality and high inflammation (PSHI) and good sleep quality and low inflammation (GSLI) markers. Scatter plot of the genetic correlation between PSQI and CRP of sleep markers. The CRP z-score shows a positive correlation with the PSHI group (R = 0.69, *P* < 2.2e-16) and a negative correlation with the GSLI group (R = −0.33, *P* < 2.2e-16). **(G)** The results obtained by measuring gene expression levels using qPCR in two distinct groups based on sleep quality. The poor sleep group (PS) consisted of three individuals with high PSQI scores, while the good sleep group (GS) included three individuals with low PSQI scores. Significant differences in gene expression were observed for the *BLVRA* and *CRISPLD2* genes.

The top eight poor and good sleep quality markers were selected according to their significance (Fig. S2). One of the top markers, *PER3*, is a representative circadian rhythm-related gene; however, other genes were also found to have an ambiguous direct relationship with sleep (Table S2). To characterize the over-represented functions of the marker genes, we performed pathway enrichment analysis for each marker group. Both markers of sleep quality were characterized by immune-related functions (Fig. 1C). Poor markers were significantly associated with pathways related to the innate immune system, leukocyte activation involved in immune response, neutrophil-mediated immunity, immunoglobulin binding, and microglial pathogen phagocytosis. Good markers were significantly associated with pathways related to T-cell receptor signaling and lymphocyte count and distribution. These results suggested an association between poor sleep quality and immune system disturbances as well as immune response abnormalities. Network analysis of the marker genes and enriched pathways showed that poor sleep quality markers formed a large cluster related to immune pathways, while good sleep quality markers formed a fully connected cluster with subclusters related to immune, chromatin modification, and cell cycle pathways (Fig. 1D).

To identify genes associated with clinical immune dysfunction, correlation analyses were performed between the marker genes and clinical parameters, including the five criteria for metabolic syndrome (waist circumference, triglyceride, high-density lipoprotein, blood pressure, and blood glucose) and C-reactive protein (CRP). As a result, ten and six genes were significantly correlated with CRP levels among the poor and good markers for sleep quality, respectively. We found that most genes (7/10) selected from the poor markers were positively correlated with CRP, indicating that increased expression of these genes was associated with poor sleep quality and a high inflammatory state (Fig. 1E, left). In contrast, all six genes selected from the good markers were negatively correlated with CRP, indicating that increased expression of these genes was associated with good sleep quality and a lower inflammatory state (Fig. 1E, right). The results supported the relationship between sleep quality and the inflammatory state of the body. The seven genes from the poor markers (*BLVRA*, *CRISPLD2*, *CYSTM1*, *FCGR1A*, *FCGR2A*, *LYL1*, and *SQOR*) were defined as markers for poor sleep quality and high inflammation (PSHI), whereas the six genes from the good markers (*ATP23*, *DBR1*, *TBCE*, *TMED6*, *GUCA1B*, and *PFN2*) were defined as markers for good sleep quality and low inflammation (GSLI) (Table S3).

To investigate the genetic influence of sleep-related genes on CRP levels, the expression of quantitative trait loci (eQTL) and associated single nucleotide polymorphisms (SNPs) in the PSHI and GSLI groups within blood cells was examined. A correlation analysis was conducted using GWAS summary statistics for sleep quality (PSQI) and CRP traits, with a focus on cis-eQTLs within 1 Mbp of the gene region. We examined the distribution of functional SNPs within the genetic regions, focusing on the eQTL relationships for each gene (Table S4). The SNP effects of the entire gene set in the PSHI and GSLI groups had a significant correlation with CRP genes (Fig. 1F). In the PSHI group, genes with sequence variations that cause increased expression had significant correlations with the CRP GWAS results, including *BLVRA* (positive SNP effects, *R* = 0.76, *P* < 2.2e-16) and *CRISPLD2* (positive SNP effects, *R* = 0.62, *P* = 1.40e-08). In the GSLI group, genes with sequence variations that cause decreased expression had significant correlations with the CRP GWAS results, including *ATP23* (negative SNP effects, *R* = −0.64, *P* < 2.2e-16) and *TBCE* (negative SNP effects, *R* = −0.025, *P* = 0.0036). To investigate whether there are significant differences in the expression of these four genetic markers based on poor sleep quality, we measured their expression levels using quantitative PCR (qPCR) in three independent individuals with high PSQI scores (PS: poor sleep group; PSQI percentile rank within the top 10%) and low PSQI scores (GS: good sleep group; PSQI percentile rank within the bottom 10%) among KDCC participants without metabolic disorders (Table S5). The results revealed significant differences in the expression of two genes within the PSHI group, depending on the quality of sleep (Fig. 1G).

Sleep deprivation can have devastating effects on physical and mental health, including obesity, diabetes, cardiovascular disease, depression, and anxiety. The relationship between sleep and the immune system is unclear, but poor sleep can lead to chronic inflammation and increased risk of infectious and inflammatory diseases.^4^ Transcriptomic analysis identified genes associated with sleep quality and inflammation markers. These genes are involved in immune response pathways and circadian rhythms. Genetic analysis revealed effector genes for sleep quality and inflammation, showing that their expression levels affect sleep quality and are correlated with CRP levels. This study emphasizes the complex interplay between genetics, sleep quality, and inflammation, highlighting the need for personalized medicine to improve sleep and suppress the development of inflammation-related diseases. Further research is needed to fully understand the underlying mechanisms.

## Author contributions

Sang-Min Park & Hyo-Jeong Ban: Conceptualization, methodology, formal analysis, visualization, and writing – original draft. Minsung Lee: Writing. Soo Yeon Kim: Validation experiments. Siwoo Lee: Resources. HeeJeong Jin: Conceptualization, writing – original draft, supervision, and project administration.

## Conflict of interests

The authors declare no conflict of interests.

## Funding

This study was supported by the “Development of Korean Medicine Original Technology for Preventive Treatment based on Integrative Big Data” grant from the 10.13039/501100003718Korea Institute of Oriental Medicine (No. KSN2023120).

## Figures and Tables

**figs1:**
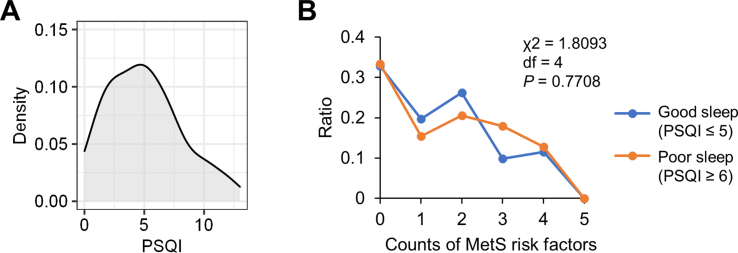


**figs2:**