# Supplementary material for: Blood transcriptomic markers associated with immune abnormalities and sleep quality
Source: Genes Dis. 2023 Sep 14;11(6):101105. doi: 10.1016/j.gendis.2023.101105 (PMC11408026; doi:10.1016/j.gendis.2023.101105)
Supplement: Multimedia component 1 [file mmc1.docx]

SUPPLEMENTARY DATA

**Blood transcriptomic markers associated with immune abnormalities and sleep quality^[[1]](#footnote-1)^**

Sang-Min Park^1†^, Hyo-Jeong Ban^2†^, Minsung Lee^1^, Soo Yeon Kim^2^, Siwoo Lee^2^, Hee-Jeong Jin^2*^

^1^ College of Pharmacy, Chungnam National University, Daejeon 34134, Republic of Korea

^2^ Korean Medicine (KM) Data Division, Korea Institute of Oriental Medicine, Daejeon 34054, Republic of Korea

^†^ These authors contributed equally to this work and share first authorship

^*^Correspondence: [hjjin@kiom.re.kr](mailto:hjjin@kiom.re.kr)

This file includes:

Supplementary Materials and Methods

Supplementary TEXT

Supplementary References

Supplementary Data legends

- Figures S1-2
- Tables S1-5

Supplementary MATERIALS AND METHODS

**Participants**

In this study, we included the same participants (n = 100) as those included in our previous study^1^ for exploring genes associated with metabolic syndrome progression. These participants were selected from the Korean Medicine Daejeon Citizen Cohort (KDCC) study, ^2^ an ongoing cohort study to assess relationships between chronic diseases and lifestyle factors. The Institutional Review Board of the Korea Institute of Oriental Medicine and the Regional Ethics Board of Dunsan Korean Medicine Hospital of Daejeon University reviewed and approved this study (IRB No. DJDSKH-17-BM-12). All participants gave their consent in writing after being fully informed.

Overall sleep quality was measured based on the respondents’ subjective responses to the Korean Pittsburgh Sleep Quality Index (PSQI). The PSQI measures sleep quality and disturbances over the previous month, summing responses to 7 sleep quality items to produce a score between 0 and 21 ^3,4^; a high PSQI score signifies a poorer sleep quality.

**Transcriptome analysis**

RNA-seq data for peripheral blood mononuclear cells (PBMCs) from the 100 participants were obtained from the Gene Expression Omnibus database (accession number GSE200744) and analyzed in R (v.4.2.2). Gene expression levels were calculated as transcripts per million (TPM) using the edgeR package (v.3.38). ^5^ Regression analysis between gene expression levels and PSQI was performed using the MASS package (v.7.3-58.1) to build linear and negative binomial regression models. Genes with low expression in more than 20% of participants with a log2(TPM+1) value of <0.5 were excluded from the analysis.

Pathway enrichment analysis for the selected marker genes was conducted using g:Profiler^6^ (<https://biit.cs.ut.ee/gprofiler/gost>), with a cutoff adjusted p-value of 0.05 and term size of 1500. The following gene sets were used in pathway enrichment analysis: 1) Gene Ontology, including molecular function and biological process; 2) Human Phenotype Ontology; and 3) biological pathways, including Kyoto Encyclopedia of Genes and Genomes, Reactome, and WikiPathways.

**Correlation analysis**

The clinical parameters of the 100 participants were obtained from our previous study, ^1^ including five criteria for metabolic syndrome (waist circumference, triglyceride, high-density lipoprotein, blood pressure, and blood glucose) and C-reactive protein (CRP), an indicator of inflammation in the body. ^7^ Pearson’s correlation analysis between the clinical parameters and gene expression levels was performed using the *cor.test* function in R (v.4.2.2).

**Network analysis**

Network analysis was performed on the results of pathway enrichment analysis for the selected marker genes. Each pathway and gene were represented by a node. The pathways were connected via links to the relevant genes, representing significance. The resulting network was visualized using Cytoscape (v.3.9.0).

**Genetic correlation between PSQI and CRP**

We performed a genome-wide association study (GWAS) on PSQI and CRP traits in the complete 2,000 participants of the KDCC study. ^2^ GWAS was performed by linear regression of each trait using PLINK 1.9 ^8^ adjusted for sex and age. The genetic correlation (Rg) of single-nucleotide polymorphism (SNP) effects (ZpsqiZcrp) between PSQI and CRP traits was estimated using Pearson’s correlation test, with summary statistics cleaned to contain sleep-related genes from RNA-seq analysis. Z-scores (effect size/standard error) of SNPs were extracted within the 1-Mbp range from six genes within the good sleep quality and low inflammation (GSLI) group and seven genes within the poor sleep quality and high inflammation (PSHI) group. To select SNPs affecting gene expression, only SNPs with significant expression quantitative trait loci (eQTL) values in blood tissues from the GTEx database v7 ^9^ were used for calculation.

**PBMC RNA extraction and quantitative PCR (qPCR)**

Total RNA from 1 × 10^6^ PBMCs was isolated with the RNeasy Plus MicroKit (Qiagen, 74034) and used for cDNA synthesis with the SuperScript™ First-Strand Synthesis System (Invitrogen, 11904018). qPCRs were performed using cDNA (10 ng), Power SYBR™ Green PCR Master Mix (Applied Biosystems™, 4367659), and primers according to the manufacturer’s instructions, and run using the Rotor-Gene Q 2plex (Qiagen, 9001620). The samples were amplified for 35 cycles as follows: 95°C for 10 s, 52°C for 30 s, and 72°C for 1 min. To analyze qPCR data, quantification was calculated using the 2^-ΔΔCt^ method with GAPDH as a housekeeping gene, and data were represented by relative fold changes. The following primers were used: ATP23, 5′-tctatcctggctgttaggaa-3′ (sense) and 5′-tcacggttttcaaagtctct-3′ (antisense); BLVRA, 5′-gagtccagcagatttctttg-3′ (sense) and 5′-gacagtgtcatggggtattc-3′ (antisense); CRISPLD2, 5′-cgaagatctttggaactctg-3′ (sense) and 5′-tttcacttttgacaccatga-3′ (antisense); GUCA1B 5′-gcttcttcaaggtcacagac-3′ (sense) and 5′-gaatgtccacttcagcttgt-3′ (antisense); PFN2 5′-tttaccaacggtttgactct-3′ (sense) and 5′-ttcccattacaaagaccaag-3′ (antisense); TBCE, 5′-gatgtcattggtcgaagagt-3′ (sense) and 5′-atagcggttcttaattgcag-3′ (antisense); and GAPDH, 5′-aacctgccaaatatgatgaca-3′ (sense) and 5′-ataccaggaaatgagcttgac-3′ (antisense).

SUPPLEMENTARY TEXT

There is no consensus on the definition of ideal sleep because it is influenced by a complex and varied set of factors, including genetics, environment, and behavior. ^10^ However, sleep deprivation can have devastating effects on animals and humans, regardless of age or sex.^11-14^ Lack of sleep has been associated with various physical health problems, such as obesity,^15^ diabetes,^16^ cardiovascular disease, and hypertension,^17^ as well as mental health issues, such as depression,^18^ anxiety,^19^ and stress.^20^ However, the relationship between sleep and the immune system is still unclear.^21-24^

Pathway enrichment analysis revealed the association between sleep quality and immune-related pathways. Sleep regulates the immune system by influencing physiological systems that can affect immune cell distribution and the production of inflammatory cytokines.^13,25^ Poor sleep can disrupt immune processes, leading to chronic inflammation and an increased risk of infectious and inflammatory diseases.^22^ Sleep deprivation-induced subclinical inflammatory status, which is marked by altered systemic and cellular inflammation, can have pathogenic implications for metabolic and cardiovascular risk factors.^23,24^ A sustained activation of the inflammatory response due to persistent sleep disturbance can harm the body, and sleep disturbance has been linked to higher CRP levels.^26,27^

We highlighted the transcriptomic marker genes that correlated with sleep quality and CRP levels in the PSHI and GSLI groups. The PSHI group included *BLVRA*, *CRISPLD2*, *CYSTM1*, *FCGR2A*, *FCGR1A*, *LYL1,* and *SQOR*, and most were associated with immune response pathways (except *SQOR* and *BLVRA*) (**Figure 1D**). Several PSHI genes have also been shown to be related to circadian rhythms and sleep disorders. *FCGR1A* and *FCGR2A* encode Fc-gamma receptors involved in both innate and adaptive immune responses and interact directly with CRP.^28^ Fc-gamma receptors may also be involved in circadian rhythms since *FCGR2A* expression levels fluctuate with light and dark cycles.^29^ *CRISPLD2* is involved in innate immunity with anti-inflammatory and anti-endotoxin effects,^30^ and its expression level also showed consistent circadian oscillations.^31^ *LYL1* is a transcription factor that controls the specification and maintenance of immune cells.^32^ In GWAS, *LYL1* was identified as a gene associated with insomnia that is directly linked to sleep quality.^33^ For the remaining three genes, *CYSTM1* is involved in innate immunity, *SQOR* responds to oxidative stress, and *BLVRA* catalyzes biliverdin; however, they do not yet have links to sleep or circadian rhythms. Given that melatonin, a hormone that regulates circadian rhythms, is essential in protecting cells from oxidative stress,^34^ *SQOR* could possibly be related to circadian rhythms as it plays a similar role.

The GSLI group included *ATP23*, *DBR1*, *GUCA1B*, *PFN2*, *TBCE*, and *TMED6*, most of which are poorly understood in terms of their relationship with the immune response and sleep. The protein encoded by *DBR1* participates in the debranching of RNA lariats, a type of circular RNA. Since the accumulation of circular RNAs is associated with the aging of the brain and the development of Parkinson’s disease, which is often accompanied by sleep disturbances,^35^ high expression of *DBR1* could contribute to sleep quality by degrading RNA lariats. *TBCE* is involved in β-tubulin folding and its mutation causes Sanjad Sakati syndrome, which was recently reported to be associated with sleep-disordered breathing.^36^ *GUCA1B* is involved in visual perception by phototransduction and has been reported to be dysregulated by blue light-induced eye damage.^37^ Since blue light suppresses melatonin secretion, thereby affecting sleep quality, *GUCA1B* expression may be related to sleep. *GUCA1B* may also play a role in immunity since its expression was found to be associated with AIDS progression in GWAS.^38^

PSHI and GSLI markers are essential in biological pathways associated with immunity and sleep. Although there are several ways to investigate the relationship between PSQI and CRP traits, we conducted a correlation analysis of the genetic influences of each trait through GWAS to examine the relationship between sleep quality and immune indicators. GWAS showed that *BLVRA* and *CRISPLD2* were the main effector genes for PSHI and that *GUCA1B*, *ATP23*, and *PFN2* were the key effector genes for GSLI. As the expression of poor markers increased, sleep quality decreased, whereas sleep quality improved as the expression of good markers increased. The association between the expression levels of the two genes in PSHI and sleep quality was validated in three independent individuals. Additionally, the genes were significantly correlated with CRP levels. Although this does not account for all genes associated with PSHI and GSLI, the amount of CRP is regulated according to the genetic effects that control sleep quality. Expression of the remaining genes may be influenced by environmental factors. CRP is used to diagnose and monitor various inflammatory reactions, such as infectious and autoimmune diseases.^39^ These results confirmed the change in the inflammatory response due to the regulation of gene expression related to sleep quality. Therefore, the risk of inflammation-related diseases is expected to increase when sleep problems occur.

In conclusion, our study provides valuable insight into the complex interplay between the genetic and environmental factors that determine sleep quality and their impact on overall health. Our findings also highlighted the importance of personalized medicine since genetic differences may affect an individual’s response to treatments aimed at improving sleep quality and reducing the risk of inflammation-related diseases. Further research is required to fully understand the molecular mechanisms underlying these relationships; however, our current study provides a solid foundation for future investigations in this field.

REFERENCES

1. Park S-M, Park M, Ban H-J, et al. Investigation of prodromal features in metabolic syndrome based on transcriptome analysis. *Genes Dis*. 2022;10(3):708-711. doi.org/10.1016/j.gendis.2022.07.021.

2. Baek Y, Seo B-N, Jeong K, Yoo H, Lee S. Lifestyle, genomic types and non-communicable diseases in Korea: a protocol for the Korean Medicine Daejeon Citizen Cohort study (KDCC). *BMJ Open*. 2020;10(4):e034499. doi.org/10.1136/bmjopen-2019-034499.

3. Mohr S, Liew C-C. The peripheral-blood transcriptome: new insights into disease and risk assessment. *Trends Mol Med*. 2007;13(10):422-432. doi.org/10.1016/j.molmed.2007.08.003.

4. Uffelmann E, Huang QQ, Munung NS, et al. Genome-wide association studies. *Nat Rev Methods Primers*. 2021;1(1):59. doi.org/10.1038/s43586-021-00056-9.

5. Robinson MD, McCarthy DJ, Smyth GK. edgeR: a Bioconductor package for differential expression analysis of digital gene expression data. *Bioinformatics*. 2010;26(1):139-140. doi.org/10.1093/bioinformatics/btp616.

6. Kuleshov MV, Jones MR, Rouillard AD, et al. Enrichr: a comprehensive gene set enrichment analysis web server 2016 update. *Nucleic Acids Res*. 2016;44(W1):W90-7. doi.org/10.1093/nar/gkw377.

7. Marnell L, Mold C, Du Clos TW. C-reactive protein: ligands, receptors and role in inflammation. *Clin Immunol*. 2005;117(2):104-111. doi.org/10.1016/j.clim.2005.08.004.

8. Chang CC, Chow CC, Tellier LC, Vattikuti S, Purcell SM, Lee JJ. Second-generation PLINK: rising to the challenge of larger and richer datasets. *Gigascience*. 2015;4(1):7. doi.org/10.1186/s13742-015-0047-8.

9. Lonsdale J, Thomas J, Salvatore M, et al. The genotype-tissue expression (GTEx) project. *Nat Genet*. 2013;45(6):580-585. doi.org/10.1038/ng.2653.

10. Chaput J-P, Dutil C, Sampasa-Kanyinga H. Sleeping hours: what is the ideal number and how does age impact this? *Nat Sci Sleep*. 2018:10:421-430. doi.org/10.2147/nss.s163071.

11. Rechtschaffen A, Gilliland MA, Bergmann BM, Winter JB. Physiological correlates of prolonged sleep deprivation in rats. *Science*. 1983;221(4606):182-184. doi.org/10.1126/science.6857280.

12. Strine TW, Chapman DP. Associations of frequent sleep insufficiency with health-related quality of life and health behaviors. *Sleep Med*. 2005;6(1):23-27. doi.org/10.1016/j.sleep.2004.06.003.

13. Irwin MR. Sleep and inflammation: partners in sickness and in health. *Nat Rev Immunol*. 2019;19(11):702-715. doi.org/10.1038/s41577-019-0190-z.

14. Grandner MA, Seixas A, Shetty S, Shenoy S. Sleep duration and diabetes risk: population trends and potential mechanisms. *Curr Diab Rep*. 2016;16(11):1-14. doi.org/10.1007/s11892-016-0805-8.

15. Prather AA, Janicki-Deverts D, Hall MH, Cohen S. Behaviorally assessed sleep and susceptibility to the common cold. *Sleep*. 2015;38(9):1353-1359. doi.org/10.5665/sleep.4968.

16. Gottlieb DJ, Punjabi NM, Newman AB, et al. Association of sleep time with diabetes mellitus and impaired glucose tolerance. *Arch Intern Med*. 2005;165(8):863-867. doi.org/10.1001/archinte.165.8.863.

17. Ayas NT, White DP, Manson JE, et al. A prospective study of sleep duration and coronary heart disease in women. *Arch Intern Med*. 2003;163(2):205-209. doi.org/10.1001/archinte.163.2.205.

18. Tsuno N, Besset A, Ritchie K. Sleep and depression. *J Clin Psychiatry*. 2005;66(10):1254-1269. doi.org/10.4088/jcp.v66n1008.

19. Alvaro PK, Roberts RM, Harris JK. A systematic review assessing bidirectionality between sleep disturbances, anxiety, and depression. *Sleep*. 2013;36(7):1059-1068. doi.org/10.5665/sleep.2810.

20. Zochil ML, Thorsteinsson EB. Exploring poor sleep, mental health, and help‐seeking intention in university students. *Aust J Psychol*. 2018;70(1):41-47. doi.org/10.1111/ajpy.12160.

21. Imeri L, Opp MR. How (and why) the immune system makes us sleep. *Nat Rev Neurosci*. 2009;10(3):199-210. doi.org/10.1038/nrn2576.

22. Garbarino S, Lanteri P, Bragazzi NL, Magnavita N, Scoditti E. Role of sleep deprivation in immune-related disease risk and outcomes. *Commun Biol*. 2021;4(1):1304. doi.org/10.1038/s42003-021-02825-4.

23. Esser N, Legrand-Poels S, Piette J, Scheen AJ, Paquot N. Inflammation as a link between obesity, metabolic syndrome and type 2 diabetes. *Diabetes Res Clin Pract*. 2014;105(2):141-150. doi.org/10.1016/j.diabres.2014.04.006.

24. Cappuccio FP, Miller MA. Sleep and cardio-metabolic disease. *Curr Cardiol Rep*. 2017;19(11):110. doi.org/10.1007/s11886-017-0916-0.

25. Irwin MR, Opp MR. Sleep health: reciprocal regulation of sleep and innate immunity. *Neuropsychopharmacology*. 2017;42(1):129-155. doi.org/10.1038/npp.2016.148.

26. Irwin MR, Olmstead R, Carroll JE. Sleep disturbance, sleep duration, and inflammation: a systematic review and meta-analysis of cohort studies and experimental sleep deprivation. *Biol Psychiatry*. 2016;80(1):40-52. doi.org/10.1016/j.biopsych.2015.05.014.

27. Besedovsky L, Lange T, Haack M. The sleep-immune crosstalk in health and disease. *Physiol Rev*. 2019;99(3):1325-1380. doi.org/10.1152/physrev.00010.2018.

28. Rosales C, Uribe-Querol E. Fc receptors: Cell activators of antibody functions. *Adv Biosci Biotechnol*. 2013;4(4):21-33. dx.doi.org/10.4236/abb.2013.44A004.

29. Sukumaran S, Xue B, Jusko WJ, Dubois DC, Almon RR. Circadian variations in gene expression in rat abdominal adipose tissue and relationship to physiology. *Physiol Genomics*. 2010;42A(2):141-52. doi.org/10.1152/physiolgenomics.00106.2010.

30. Zhang S, Pei L, Qu J, et al. CRISPLD2 attenuates pro-inflammatory cytokines production in HMGB1-stimulated monocytes and septic mice. *Am J Transl Res*. 2021;13(5):4080-4091.

31. Wittenbrink N, Ananthasubramaniam B, Münch M, et al. High-accuracy determination of internal circadian time from a single blood sample. *J Clin Invest*. 2018;128(9):3826-3839. doi.org/10.1172/JCI120874.

32. Zohren F, Souroullas GP, Luo M, et al. The transcription factor Lyl-1 regulates lymphoid specification and the maintenance of early T lineage progenitors. *Nat Immunol*. 2012;13(8):761-769. doi.org/10.1038/ni.2365.

33. Sun H, Zhang J, Ma Y, Liu J. Integrative genomics analysis identifies five promising genes implicated in insomnia risk based on multiple omics datasets. *Biosci Rep*. 2020;40(9):BSR20201084. doi.org/10.1042/BSR20201084.

34. Reiter RJ, Tan DX, Osuna C, Gitto E. Actions of melatonin in the reduction of oxidative stress. A review. *J Biomed Sci*. 2000;7(6):444-458. doi.org/10.1007/BF02253360.

35. Doxakis E. Insights into the multifaceted role of circular RNAs: implications for Parkinson's disease pathogenesis and diagnosis. *NPJ Parkinsons Dis*. Jan 10 2022;8(1):7. doi.org/10.1038/s41531-021-00265-9.

36. Al-Yaarubi S, Al-Abri AS, Al-Kindi H, Al-Abri M, Naz T, Khater D. Sanjad Sakati syndrome and sleep-disordered breathing: an undisclosed association. *Sleep Breath*. Jun 2022;26(2):815-821. doi.org/10.1007/s11325-021-02463-4.

37. Ouyang XL, Chen BY, Xie YF, et al. Whole transcriptome analysis on blue light-induced eye damage. *Int J Ophthalmol*. 2020;13(8):1210-1222. doi.org/10.18240/ijo.2020.08.06.

38. Spadoni JL, Rucart P, Le Clerc S, et al. Identification of Genes Whose Expression Profile Is Associated with Non-Progression towards AIDS Using eQTLs. *PLoS One*. 2015;10(9):e0136989. doi.org/10.1371/journal.pone.0136989.

39. Ansar W, Ghosh S. Inflammation and inflammatory diseases, markers, and mediators: Role of CRP in some inflammatory diseases. In: Ansar W, Ghosh S, eds. *Biology of C reactive Protein in Health and Disease*. 1st ed. Springer New Delhi; 2016:67-107.

**Supplementary Data legends**

Figure S1. Relationship between MetS risk factors and PSQI score. (A) The PSQI scores of the participants ranged from 0 to 13, with an average of 5.02. We included 100 individuals who had been examined for the association between metabolic syndrome and genes. (B) Before conducting the study on sleep quality, we checked for differences in the distribution of participants with metabolic syndrome according to sleep quality; however, no significant difference was observed.

Figure S2. The top eight poor and good markers for sleep quality according to their significance.

Table S1. Regression analysis results for the markers of poor and good sleep quality.

Table S2. The top eight poor or good markers for sleep quality with information obtained from GeneCard (<https://www.genecards.org/>).

Table S3. The markers for poor sleep quality and high inflammation (PSHI) and good sleep quality and low inflammation (GSLI) with information obtained from GeneCard (<https://www.genecards.org/>).

Table S4. Distribution of blood expression quantitative trait loci (eQTL) single-nucleotide polymorphisms (SNPs) in functional regions within the 1 Mbp range of sleep genes. The correlation coefficient (R) and statistical significance (p-value) for the SNP effects of PSQI and CRP are confirmed.

Table S5. Demographic characteristics and metabolic measures in validation samples.

1. CRP, C-reactive protein; eQTL, expression quantitative trait loci; GS, good sleep group; GSLI, good sleep quality and low inflammation; GWAS, genome-wide association study; PS, poor sleep group; PSHI, poor sleep quality and high inflammation; PSQI, Pittsburgh Sleep Quality Index; qPCR, quantitative PCR; SNP, single-nucleotide polymorphism. [↑](#footnote-ref-1)
